# Running Marathons in High School: A 5-Year Review of Injury in a Structured Training Program

**DOI:** 10.3390/ijerph20054426

**Published:** 2023-03-01

**Authors:** Mary A. Kennedy, Lauren V. Fortington, Matt Penney, Nicolas H. Hart, Pierre A. d’Hemecourt, Dai Sugimoto

**Affiliations:** 1 School of Medical and Health Sciences, Edith Cowan University, Joondalup, WA 6027, Australia; 2 Nutrition and Health Innovation Research Institute, Edith Cowan University, Joondalup, WA 6027, Australia; 3 Advanced Sports Therapy, Wellesley, MA 02481, USA; 4 Sports Rehabilitation Unlimited, Middleton, MA 01949, USA; 5 Caring Futures Institute, College of Nursing and Health Sciences, Flinders University, Adelaide, SA 5000, Australia; 6 Human Performance Research Centre, School of Sport, Exercise and Rehabilitation, University of Technology (UTS), Sydney, NSW 2021, Australia; 7 Institute for Health Research, University of Notre Dame Australia, Perth, WA 6027, Australia; 8 Division of Sports Medicine, Department of Orthopaedics, Boston Children’s Hospital, Waltham, MA 02115, USA; 9 Harvard Medical School, Harvard University, Boston, MA 02115, USA; 10 The Micheli Center for Sports Injury Prevention, Boston Children’s Hospital, Waltham, MA 02453, USA; 11 Faculty of Sport Sciences, Waseda University, Tokyo 202-0021, Japan

**Keywords:** sports medicine, athletic injuries, high school, running

## Abstract

Objective: The aim in this study was to quantify the number, nature, and severity of injuries sustained by male and female high school students who took part in a running training program that culminated in the completion of a half or full marathon. Design: This study is a retrospective clinical audit. Methods: Injury reports from high school students (grades 9–12) who participated in a half or full marathon 30-week progressive training program comprising four training days per week (three running days and one cross-training day) were reviewed. The number of runners completing a marathon, together with the number, nature, severity of injuries, and treatment types, as reported to the program physiotherapist, were the main outcome measures. Results: Program completion was 96% (*n* = 448/469). Of all participants, 186 (39.6%) were injured, with 14 withdrawing from the program due to injury. For those who completed a marathon, 172 (38%) reported 205 musculoskeletal injuries (age of injured runners: 16.3 ± 1.1 years; 88 girls (51.2%) and 84 boys (48.8%)). More than half (*n* = 113, 55.1%) of the reported injuries were soft tissue injuries. Most injuries were localized to the lower leg (*n* = 88, 42.9%) and were of a minor nature (*n* = 181, 90%), requiring only 1–2 treatments. Conclusions: There was a low number of relatively minor injuries for high school participants taking part in a graduated and supervised marathon training program. The injury definition was conservative (i.e., any attendance to physiotherapist) and the relative severity of injuries was minor (i.e., requiring 1–2 treatment sessions). Overall, these results do not support a need to restrict high school students from taking part in marathon running, though continued emphasis on graduated program development and close supervision of young participants is recommended.

## 1. Introduction

Physical inactivity is one of the world’s greatest public health challenges [1]. This issue is especially concerning among children, as research suggests that children who are inactive are more likely to be less physically active in adulthood [2]. To counter this undesirable phenomenon, initiatives for students to engage with a wide variety of traditional and nontraditional sporting activities have been developed. One of the approaches to promote physical activity in middle school and high school students is noncompetitive marathon training, such as the renowned Students Run LA (Los Angeles) program [3]. The Students Run LA program has inspired the development of similar programs in other cities across the United States designed to encourage physical activity in those who are less interested or unable to participate in the traditional competitive high school or club sports. Alongside the growth of these programs, however, has been continuing concern and debate on the safety of adolescents participating in a long-distance running such as marathons and other endurance-type events [4,5,6].

Marathon running is perceived as an “extreme” running event that requires a high level of physical and psychological maturity [7]. Adequate preparation for the event requires months of training, combined with quality nutrition and sleep schedules [7]. A marathon consists of 26.2 miles (41.2 km) across various environmental conditions; thus, training and completing the event requires substantial mental and physical discipline. There is limited evidence on the safety of child and adolescent participation in marathon events [8]. A position statement published in 2003 by Rice and Wainewski remains one of the most comprehensive resources on adolescent marathon running [4]. Rice and Wainewski recommended against allowing adolescents to participate in such events, citing potential adverse effects of distance running on their growth, development and long-term health [4]. The potential risks of developing overuse injuries, along with long-term negative health implications such as growth plate injuries and adult-onset arthritis, were speculated as major concerns in Rice and Wainewski’s report.

In response to the Rice and Wisniewski statement, an expert panel consisting of an interdisciplinary team of physicians, researchers, and specialists in adolescent physical activity was convened in 2006 to share opinions on safety of adolescents in marathon participation [4]. One theme derived by the panel was the “need of a great deal more research information” on the topic before its safety could be fairly evaluated and evidence based consensus be generated [4]. Over a decade later, objective data regarding the safety of marathon running in adolescents remains scarce. Despite the lack of agreement on safety, including what the minimum age to begin marathon running should be, there has been a notable increase in marathon participation by adolescents in the last 10 years. It is therefore important to address gaps in evidence and potentially identify specific risks and benefits for this group of participants to ensure age-appropriate policy and rules that facilitate safe experiences in marathon running.

To address some of these evidence gaps, the primary aim of our study was to identify the number, nature, and severity of injuries sustained by adolescents who took part in an organized high school marathon training program. Additionally, because growth and maturation between female and male adolescents differ, an exploratory examination of differences in outcomes by sex was also undertaken as a secondary aim. With knowledge on the injuries sustained, a better understanding of adolescent marathon runners can be made available to inform future strategies that promote safe participation, and the development of future programs that prevent injury.

## 2. Methods

### 2.1. Study Design

This is a retrospective clinical audit of prospectively collected data on injury presentations. Ethics approval to collect and report data was obtained through the Boston Children’s Hospital Institutional Review Board. Injury data were collected by licensed physiotherapists at the Advanced Sports Therapy physiotherapy clinic (USA). Treatment decisions were made independently from the organization of the distance running program. The current study was performed prior to the COVID-19 pandemic outbreak.

### 2.2. Participants

High school students (grades 9–12) volunteered to take part in the marathon training program. All students were welcome to participate if they could commit to attending weekly practices. There were no try-outs or fitness/performance expectations; participants did not need prior running experience; and nobody was excluded from the team due to their performance. Students were required to have a pediatrician’s medical clearance prior to participation. Parental consent was provided for all participants younger than 18 years of age. Individual consent was provided by participants 18 years of age or older.

### 2.3. Program Structure

The DREAMFAR High School Marathon program (DREAMFAR) is a noncompetitive marathon-training program available to students from participating high schools in the Greater Boston area (Boston, MA, USA). In existence since 2008, over 600 students have successfully trained to complete a half or full marathon. Information sessions are held at participating schools to invite students to participate. All practices are supervised and managed in a noncompetitive manner, with an emphasis placed on fun and participation. The program has a “train-the-trainer” model, whereby high school staff members (adults) volunteer to be a school leader. These leaders take part in an orientation session and receive support and training throughout the marathon season from the program staff. Leaders establish weekly meeting schedules, create safe training routes around the school and community, and support the students through prescribed workouts. Separate to the leaders, adult mentors are recruited through various community networks to train with students during longer weekend runs. Mentors are required to undergo criminal background checks and complete a program orientation and training to learn mentorship “best practices” prior to participation in the program. Students and mentors are grouped into “running families” (~1:2 mentor:student ratio) to ensure all students have support on the road. If a student has special circumstances that requires constant supervision, an individual mentor is assigned. The school leaders, together with adult mentors and student participants, also receive education regarding injury recognition, prevention, and management throughout the season by the program’s physiotherapy provider.

### 2.4. Training Method

The marathon training program was modeled on best practice evidence at the time [6]. Three school-based training days each week were delivered after school at participating high schools (2 days running; 1-day cross-training) (Table S1). Additionally, on Saturdays, all students (hereafter referred to as participants) met at a central location to complete a team long run together. Total mileage per week progressed conservatively, ensuring total mileage did not increase more than 10% from the week before. The program started at 2–4 miles per week and progressed to 35 miles per week over the 30-week training program [9,10]. The schedule also included one nonrunning cross-training day each week to complement the running progression with mental and physical “active rest”. The cross-training component of the program was not individualized. It was delivered in a group format and supervised by the school leader. The cross-training program was designed by the team physiotherapist at AST. It incorporated a mix of resistance training, flexibility (yoga/stretching/foam rolling), and nonrunning cardio activities that included utilizing equipment at school or at a local gym/fitness center (e.g., stationary bicycle, rowing ergometer, elliptical/arc trainer machines, or pool swimming). Activities were selected to address muscles specifically utilized during the running motion, with attention to injury prevention/load tolerance movements and exercise. Students were taught the fundamental principles of progressive overload and encouraged to increase workload when appropriate. Periodically, cross-training sessions were supplemented with discussions from local experts on relevant marathon training topics (e.g., stress management, nutrition, and sneaker fitting).

The program (“marathon season”) operated for a total of 30 weeks, from October through to May, each school year. Long weekend runs began in November and continued through to the main event—a half or full marathon, held in early May. Three races (5K, 10K, and half marathon) were strategically scheduled throughout the training period to maintain participants’ training interests and to provide race experience prior to the main event. All students were expected to attend at least 75% of scheduled training sessions in order to be eligible to run in the main event.

### 2.5. Data

The sex and age of all participants were collected from intake forms completed at the beginning of each season. Participant age was calculated based on the date of the marathon for each season as a reference point. Race completion statistics were collected via the marathon organization’s online results database. All information was compiled by the program staff, de-identified, and subsequently shared with the study team.

### 2.6. Injury Tracking and Definitions

A running-related injury was defined as any self-reported complaint from a participant to the physiotherapist. Initially, notifications of injuries were given to the student mentor or school leader, who, in turn, notified an AST physiotherapist through email to arrange an appointment for injury assessment. An appointment was scheduled within one week of initial notification. Following assessment and diagnosis, a physiotherapist provided treatment or referred the participant on to a local orthopedist if necessary. During runs ≥16 miles, a designated physiotherapist was on-site at the training run to allow students to immediately report a potential injury. Follow-up appointments were scheduled if necessary. Six physiotherapists treated the participants over the course of the study. Their notes and diagnosis were reviewed by the lead physiotherapist and author (MP) at regular weekly intervals. Any concerns were clarified with the consulting physiotherapist, recorded in the main clinical database, and flagged as a DREAMFAR participant. These records were marked for review at the time of the clinical audit. Relevant information was compiled for each participant, de-identified using previously assigned codes, and shared with the study team.

The main variables of interest were the number of participants referred to, and subsequently treated by, the physiotherapy team, together with specific details of the injuries including:

- Injury types: joint injury (e.g., ankle ligament sprain); soft tissue injury (e.g., calf muscle strain); bone injury (e.g., tibial stress reaction).
- Injured body parts (lower limb only): spine, hip/thigh, knee, lower leg, and ankle/foot.
- Treatment frequency: number of treatment sessions provided by physiotherapists. This variable was used as an indicator for severity of the injury.
- Treatment types: consultation, running modification, orthotics/brace/footwear, physiotherapy (i.e., therapeutic exercise and manual therapy modalities) and combinations of these treatments.

### 2.7. Statistical Analysis

Descriptive statistics were used to analyze the main outcome measures for the primary aim of the study. Mean, standard deviation, and 95% confidence interval (CI) values were analyzed to describe continuous variables such as number of participants referred to and treated by the physiotherapy team. To compare the number of participants referred to and treated by the physiotherapy team by sex, an independent *t*-test was used when this variable was normally distributed. Mann–Whitney U test was used when non-normal data distribution were found. The status of normality was examined using the Shapiro–Wilk test. For categorical variables, the frequency of specific variables, including injury types and injured body parts, were summed and converted to percentages (%). Analysis considered participant sex (male/female) using a chi-square (*x*^2^) analysis, with comparisons performed to evaluate percentage (%) differences in injury types and injured body parts, between groups, with *p* ≤ 0.05 used as a critical statistical value. All data were initially provided to the research team as a Microsoft Excel (Microsoft Corporation, Redmond, DC, USA) spreadsheet, and subsequently converted for analysis in SPSS (Version 23, SPSS Inc., Chicago, IL, USA).

## 3. Results

**Figure 1:**
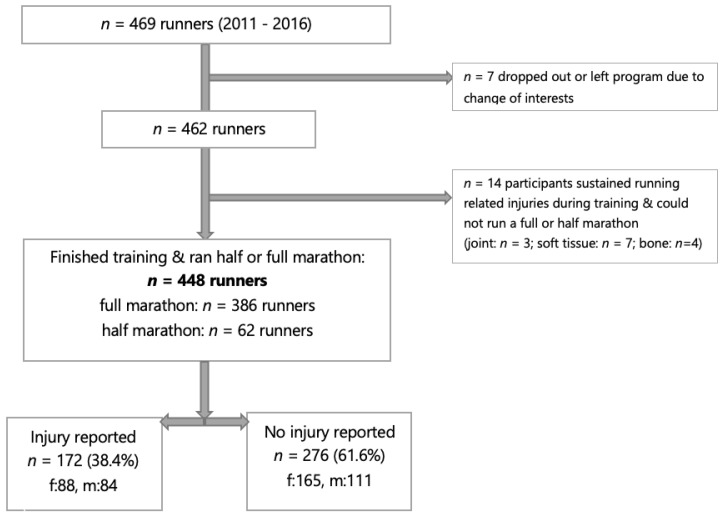
Participants who entered and completed the program, with or without injury.

Over the 5-year period of this study, 469 high school students participated in the DREAMFAR program. Seven participants chose to leave the program due to a change in interest. Fourteen participants dropped out of the program due to injury (3% of all participants). In total, 448 participants (253 girls and 195 boys) completed the full training season, which culminated with participation in a half or full marathon (Figure 1).

For all registered participants, the proportion with injuries was 39.6% (186/469). Of those who completed the program, 226 musculoskeletal injuries were reported in 172 participants (age: 16.3 ± 1.1 years; 88 girls (88/172, 51.2%) and 84 boys (84/172, 48.8%)). Twenty-nine participants reported multiple injuries during the season. A total of 143 participants reported a single injury: 143/172, 83.1% (girls: 72/143, 50.4%; boys: 71/143, 49.6%); 25 participants reported 2 injuries: 25/172 14.5% (girls: 14/25, 56.0%; boys: 11/25, 44.0%); 4 participants reported 3 injuries: 4/172, 2.3% (girls: 2/4, 50.0%; boys: 2/4, 50.0%).

Two-thirds of injuries (*n* = 154, 68.1%) were soft tissue injuries (Table 1), with 53 to the ankle or foot (23.4% of total injuries) and 48 to the lower leg (21.2%). For female runners, there were slightly more soft tissue injuries to the lower leg (*n* = 26, 21.7%) than the foot/ankle (24, 20%); while for male runners, the foot/ankle (29, 27.4%) had slightly more soft tissue injuries than the lower leg (22, 20.8%). The types of injuries sustained and the body parts injured did not differ by sex (injury type *x*^2^ df = 2, *n* = 205, *p* = 0.779; body part injured *x*^2^ df = 4, *n* = 205, *p* = 0.167).

A mean of 1.8 ± 1.6 (95% CI: 1.5, 2.0) treatment sessions were required per participant and 1.9 ± 1.7 (95% CI: 1.6, 2.1) sessions per injury. There were no reported differences in the frequency of treatment by sex or for injuries (injury treatment session by sex: independent *t*-test (df = 203, *n* = 205 *p* = 0.293)). The most common treatment types reported based on the number of injuries (*n* = 226) are reported in Table 2.

## 4. Discussion

To the best of our knowledge, this study is the first to prospectively document injuries sustained by a cohort of adolescents who took part in a marathon training program. The proportion of injuries reported in our study aligns with the lower range of injuries reported in other studies that involved adult novice running groups, starting from 26% of 629 runners in an 8-week training program (to run a 4-mile event) to as many as 85% of 63 runners in an 18–20-month marathon training program [11,12,13]. Novice runners reportedly have a higher risk of injury (17.8 (95% CI 16.7–19.1) per 1000 h of running) than recreational runners (7.7 (95% CI 6.9–8.7)) [14]. The conservative graduated training approach applied in the DREAMFAR marathon training program is likely to have contributed to the relatively low injury proportion reported. However, this should be confirmed with individual exposure measures considering the volume and intensity of training.

Importantly, the impact of the reported injuries on participants’ ability to continue training was considered minimal. Four in every five injuries reported in our study required only 1–2 treatment sessions to return to running. When compared with high school runners engaged in a competitive cross-country program, our cohort reported injuries that were less severe in terms of treatment needs [15]. This low severity was despite the nonspecific injury definition used and encouragement for participants to report any complaint, no matter how minor it seemed, as safety was the foundation of the program’s philosophy. In addition, the prevalence and severity of injuries from this running program were generally lower and had less impact than the prevalence and severity of injury in other high school sports [16,17]. In particular, arguments concerning excessive or acute damage to the adolescent joint structure from marathon running appear to be unfounded. In total, 19% of all injuries reported in this study were related to bone or skeletal injuries, including epiphyseal/apophyseal injuries and stress reaction/fractures.

Anecdotally, resistance to adolescent marathon participation has stemmed from the supposed vulnerability of growth plate injuries in skeletally immature athletes [4]. This rationale reflects traditional approaches to understanding running injury causation, which have been largely focused on individuals, particularly in relation to biomechanical, load, and impact forces [18]. The premise from this previous work led to a general acceptance that repetitive loads sustained in distance running would be detrimental to growing bodies. Despite this, the incidence, frequency, severity, and outcome of physeal injuries have been inconsistently documented in the epidemiological literature [19]. Of note, we report a particularly low occurrence of skeletal injuries (stress reaction/fracture, avulsion fracture, and physeal injury) over the five years of this study. A systematic review looking into running-related injury found no strong evidence of age-related risks with distance running [20], though, admittedly, few studies have considered younger populations. While nine studies reported a statistically significant association of injury with age, five of these studies did not identify a clear finding on the direction of the age effect being toward older or younger runners [20]. A recent review of mixed-distance running identified a small number of studies had included adolescents, but only one study of cross-country running had focused on younger runners [21]. Therefore, more research that includes younger distance-running athletes is needed.

Engaging adolescents in high school sports is an important goal; yet, many adolescents are not able, or do not wish, to participate in highly competitive settings [22]. The DREAMFAR marathon program, with an inclusive, noncompetitive nature, targeted students who would otherwise be left out of athletics programs. Almost every student (98%) who started in the program was able to complete their event (half or full marathon), mirroring those of other high school programs across the USA [3]. Overall, the results lend strong support to the delivery of programs that enroll students based on their interests and seek to prepare them both physically and emotionally for participating in regular training and working to complete a marathon [8].

A strength of this study was its systematic, consistent data collection structure. The data collection was consistent for the five-year program, with author MP supervising and organizing the collection of information throughout this period. All injury assessments were performed by physiotherapists at the clinic to which author MP belongs. In this clinic, all physiotherapy staff had completed a masters or doctorate level of education. All staff had experience exclusive to sports and orthopedic physiotherapy and utilized their clinical decision making to determine a specific physiotherapy diagnosis, prognosis, and intervention. In cases where diagnostic imaging and/or injury diagnosis confirmation was indicated, each staff member referred the athlete to a local orthopedic specialist, though these referrals were rarely needed.

Additionally, as with all descriptive studies of injury, the awareness, recognition, and communication of the injury event is vital to the accuracy of the data collection. In the DREAMFAR program, team leaders, program mentors, and all participants were instructed on several occasions about nutrition and hydration, climate awareness, proper equipment, injury prevention, and symptom recognition and management strategies and techniques by one of the authors (MP) and other professionals specifically trained and experienced in their relevant fields.

### Limitations

There are several limitations to this study. First, while free “insurance-blind” injury assessments were available to participants of the DREAMFAR program, some participants might have chosen to seek treatment elsewhere. While there is no guarantee all injuries were captured, we are confident that the most serious were included, with knowledge that only 14 (3%) participants dropped out of the running program due to injury. Second, we were unable to accurately calculate exposure in hours; therefore, injury rates were not calculated, which limits the comparability of results to those of other studies. Third, this study reports on one running program in one region, limiting its generalizability. Replication studies are encouraged to be published as being able to provide adolescent distance runners, as well as coaches and medical staff, with reliable information on injury and injury prevention is important. Fourth, while our study did not find a high proportion of lower limb bone injuries, there are additional factors to be considered before active promotion of marathon running in adolescents. Fifth, we did not have anthropometric data on participants, and participants were of a small age range, between 14 and 18 years. Unlike other studies of injuries in high school aged athletes [23], we did not find a significant difference between injured boys and girls for any of the measured variables (injury severity, type, or). This may have been due to a lack of anthropometric parameters. Finally, it was not possible to identify important physical markers in the participants of this study, such as skeletal maturity or joint hypermobility, which could provide important differential points for future consideration. Furthermore, future studies are warranted to longitudinally track key physical markers and joint-related variables so that a long-term effect of long-distance running will be verified.

## 5. Conclusions

Our study supports the safety of long-distance running training and the completion of a full or half marathon in adolescent, novice runners. Overall, 40% of marathon completers sustained musculoskeletal injuries. Most of these injuries were minor, requiring 1–2 treatment sessions only. Directly comparing results with those of other studies was limited because of the different definitions used and different age groups included. However, the results suggest that the pattern of injury is similar to that seen in adult populations of novice runners. The results do not support the need to restrict adolescent marathon programs, though a continued emphasis on conservative, graduated, and supervised training programs is recommended. Future studies need to employ a longitudinal study design to monitor the long-term effects.

## Figures and Tables

**Table 1:** Injuries by sex, body part, and injury type for the DREAMFAR training program.

Combined	Ankle/Foot		Lower Leg		Knee		Hip/Thigh		Spine		Total Injury Type	Total % Injury Type *
	*n*	% *	*n*	%	*n*	%	*n*	%	*n*	%		
Joint	11	15.7	0	0.0	33	56.9	4	12.5	11	100.0	59	26.1
Soft Tissue	53	75.7	48	87.3	25	43.1	28	87.5	0	0.0	154	68.1
Bone	6	8.6	7	12.7	0	0.0	0	0.0	0	0.0	13	5.8
Total # Body Parts	70	-	55	-	58	-	32	-	11	-	226	100.0
% Body Part	31.0	-	24.3	-	25.7	-	14.2	-	4.9	-	100	
Girls												
Joint	5	14.7	0	0.0	18	54.5	1	6.7	8	100.0	32	26.7
Soft Tissue	24	70.6	26	86.7	15	45.5	14	93.3	0	0.0	79	65.8
Bone	5	14.7	4	13.3	0	0.0	0	0.0	0	0.0	9	7.5
Total # Body Part	34	-	30	-	33	-	15	-	8	-	120	100.0
% Body Part	28.3	-	25.0	-	27.5	-	12.5	-	6.7	-	100	
Boys												
Joint	6	16.7	0	0.0	15	60.0	3	17.6	3	100.0	27	25.5
Soft Tissue	29	80.6	22	88.0	10	40.0	14	82.4	0	0.0	75	70.8
Bone	1	2.8	3	12.0	0	0.0	0	0.0	0	0.0	4	3.8
Total # Body Part	36	-	25	-	25	-	17	-	3	-	106	100.0
% Body Part	34.0	-	23.6	-	23.6	-	16.0	-	2.8	-	100	

* column percentage (and of injury nature for each body part).

**Table 2:** Frequency and type of treatment reported in DREAMFAR program.

Treatment Type	*n*	%
Physiotherapy	67	29.6
Combined physiotherapy and running modification	57	25.2
Orthotics/bracing/footwear	30	13.4
Combined physiotherapy and orthotics/bracing/footwear	27	11.9
Combined physiotherapy and running modification and orthotics/bracing/footwear	24	10.6
Referral to orthopedic specialist	11	4.9
Referral to orthopedic specialist and running modification	10	4.4

## Data Availability

Most of the relevant data were presented in the manuscript. The data are not publicly available due to the privacy of participants.

## Supplementary Materials

The following supporting information can be downloaded at: https://www.mdpi.com/article/10.3390/ijerph20054426/s1, Table S1: Sample training schedule.
