# Supplementary material for: Running Marathons in High School: A 5-Year Review of Injury in a Structured Training Program
Source: Int J Environ Res Public Health. 2023 Mar 1;20(5):4426. doi: 10.3390/ijerph20054426 (PMC10001535; doi:10.3390/ijerph20054426)
Supplement: Supplementary file 1 [file ijerph-20-04426-s001.zip › ijerph-2145682-supplementary.pdf]

Supplemental Table S1. Sample training schedule

**Week** number	**Weekday Runs (2 days per week)**± miles	minutes	**Long Run (miles)**
1 – 5	1 – 2.5	10 – 35	0
6 – 10*	2 - 4	25 - 50	4 - 6
11 – 15	2.5 - 5	30 - 60	6 – 10
16 – 20*	3 - 6	35 - 60	10 - 14
21 – 27*	3 - 7	35 - 75	14 - 20
28 – 30# (taper)	3 - 5	35 - 50	8 - 12

**Includes participation in an organized road race: 5K (week 6-10), 10K (week 16-20), half-marathon (week 21-27)*

#*Full marathon at the end of week 30*

±*A third training day is scheduled each week that consists of a strength training program co-designed by the team's coach and physical therapist*
